# Supplementary material for: Content-rich biological network constructed by mining PubMed abstracts
Source: BMC Bioinformatics. 2004 Oct 8;5:147. doi: 10.1186/1471-2105-5-147 (PMC528731; doi:10.1186/1471-2105-5-147)
Supplement: Additional File 2 — The original results of the above study (non-essential files are deleted to keep the file size under the limit set by BMC bioinformatics). [file 1471-2105-5-147-S2.bz2 › chilibotAdditionalFile2/dip40/356ID9062194E4998/html/IL1R1_IL1RN.html]

 


 **IL1R1** and **IL1RN** 
  
Found 1322 abstracts in PubMed, retrieved 40.  
 

 What does Google say? 
 PDF only 
| .edu only 

---

**Interactive relationship** (e.g. stimulation, inhibition, etc)

**Non-interactive relationship** (e.g. studied together, co-existance, homology, etc.)

- When recombinant IL1ra  [ **IL1RN** ]   [ **IL1R1** ]  was titrated into the assay, phagocytosis of the opsonized RBC was significantly inhibited p=0.002.  Ref: 12829590 Blood< MedlineTA>Blood,
- In this study, we injected recombinant adeno associated virus rAAV vectors expressing the interleukin 1 receptor  [ **IL1R1** ]  antagonist rAAV IL 1ra  [ **IL1RN** ]  into the cortex of rats experiencing transient cerebral ischemia.  Ref: 12858036 Neuroreport, 2003
- Interleukin 1 receptor  [ **IL1R1** ]  antagonist IL 1Ra  [ **IL1RN** ]  modulates the activity of IL 1, a cytokine associated with inflammatory response.  Ref: 12714833 J Thromb Thrombolysis, 2002
- The secreted form of the interleukin 1 receptor  [ **IL1R1** ]  antagonist IL 1Ra  [ **IL1RN** ]  is an acute phase protein intervening in the counterregulation of inflammatory processes.  Ref: 12716739 Diabetes< MedlineTA>Diabetes, 2002
- Interleukin 1 receptor  [ **IL1R1** ]  antagonist IL 1RA  [ **IL1RN** ]  polymorphism in women with cervical cancer.  Ref: 12820354 Anticancer Res< MedlineTA>Anticancer Res, 2002
- The decline in phagocytosis was correlated with a significant production of IL1 receptor antagonist IL1ra  [ **IL1RN** ]   [ **IL1R1** ]  by both monocytes p=0.036 and granulocytes p=0.0002 within 4 hours.  Ref: 12829590 Blood< MedlineTA>Blood, 2002
- Interleukin 1 Receptor  [ **IL1R1** ]  Antagonist IL 1RN  [ **IL1RN** ]  Genotype Modulates the Replicative Capacity of Human Endothelial Cells.  Ref: 12764021 Circ Res< MedlineTA>Circ Res, 2002
- 5 Neutrophil migration induced by LPS or BK was reduced by interleukin 1 receptor  [ **IL1R1** ]  antagonist IL 1ra  [ **IL1RN** ]  1 mg kg 1, sheep anti rat TNF serum anti TNF serum 0.3 ml cavity 1, and the nonspecific selectin inhibitor fucoidin 10 mg kg 1.  Ref: 12770932 Br J Pharmacol, 2003
- Using colonic explant cultures from 132 IBD and control subjects, we examined tumour necrosis factor alpha TNF alpha, interleukin IL 1 and interleukin 1 receptor  [ **IL1R1** ]  antagonist IL 1RA  [ **IL1RN** ]  production in vitro in response to bacterial activators.  Ref: 12823284 Clin Exp Immunol, 2003
- The authors wished to evaluate whether serum levels of interleukin 1 receptor  [ **IL1R1** ]  agonist IL 1Ra  [ **IL1RN** ]  and tumor necrosis factor alpha TNF alpha are elevated in children with LCH and decrease after chemotherapy.PATIENTS AND METHODSCirculating levels of IL 1Ra  [ **IL1RN** ]  and TNF alpha were measured in 23 and 8 children with LCH, respectively, and 7 pediatric controls using commercially available ELISA kits.  Ref: 12794527 J Pediatr Hematol Oncol, 2003
- METHODS Expression of interleukin 1 receptor  [ **IL1R1** ]  antagonist IL 1Ra  [ **IL1RN** ]  and soluble tumor necrosis factor receptor sTNFR was examined by ELISA in cells from freshly isolated synovial fluids SF and synovial tissues ST from patients with OA or RA, either left untreated or treated with IFN a.  Ref: 12734885 J Rheumatol, 2003
- The aims of the study were firstly, to measure the interleukin 1 receptor  [ **IL1R1** ]  antagonist IL 1ra  [ **IL1RN** ]  and tumour necrosis factor alpha soluble receptors p55 TNFsr1 and p75 TNFsr2 in plasma and urine, and secondly to test their response to acutely induced hyperglycaemia in Type 1 diabetes mellitus DM 1.  Ref: 12679866 Diabetologia, 2003
- Cytokines interleukin 8 IL 8, interleukin 10 IL 10, interleukin 1 receptor  [ **IL1R1** ]  antagonist IL 1ra  [ **IL1RN** ]  and tumour necrosis factor soluble receptor 2 TNFsr2 and the leucocyte adhesion molecules L selectin, HLA DR, CD18 and CD11b were determined.  Ref: 12842763 Cytokine, 2003
- Interleukin 1 receptor  [ **IL1R1** ]  antagonist IL 1ra  [ **IL1RN** ]  is an important anti inflammatory cytokine that blocks all known actions of IL 1 and markedly protects against experimentally induced ischemic, excitotoxic, and traumatic brain insults.  Ref: 12878687 J Neurosci, 2003
- OBJECTIVE Our purpose was to determine the clinical significance of interleukin 1 receptor  [ **IL1R1** ]  antagonist IL 1ra  [ **IL1RN** ] , which is an endogenous inhibitor cytokine of IL 1, in patients with cervical carcinoma.  Ref: 12694657 Gynecol Oncol, 2003
- In this study, we examined the relationship between the interleukin 1 receptor  [ **IL1R1** ]  antagonist variable number tandem repeat allele 2 IL 1RN  [ **IL1RN** ] \*2\*2 and EC replicative capacity.  Ref: 12764021 Circ Res< MedlineTA>Circ Res, 2003
- In addition, a combination of alleles influencing interleukin 1 receptor  [ **IL1R1** ]  antagonist IL1Ra  [ **IL1RN** ]   [ **IL1R1** ]  and TGFbeta1 production A2 allele for  **IL1RN**  and rare C allele in codon 25 of TGFB1 gene was associated with non responsiveness p< ; 0.05.  Ref: 12759288 Ann Rheum Dis< MedlineTA>Ann Rheum Dis, 2003
- Taken together, these results suggest that at least one mechanism of action of anti D is via the production of the anti inflammatory cytokine IL1ra  [ **IL1RN** ]   [ **IL1R1** ]  which can negatively regulate the ability of leukocytes to phagocytose opsonized cells.  Ref: 12829590 Blood< MedlineTA>Blood, 2003
- Median clearance values ranged between 25 and 54 ml min for interleukin 1 receptor  [ **IL1R1** ]  antagonist IL 1ra  [ **IL1RN** ] , 23 and 42 ml min for IL 6 and 15 and 28 ml min for TNF alpha.  Ref: 12808174 Nephrol Dial Transplant< MedlineTA>Nephrol Dial Transplant, 2003
- All subjects underwent plasma insulin, glucose, interleukin 6 IL 6, interleukin 10 IL 10, interleukin 1beta IL 1beta, interleukin 1 receptor  [ **IL1R1** ]  antagonist IL 1ra  [ **IL1RN** ] , C reactive protein, and tumor necrosis factor alpha TNF alpha levels determination.  Ref: 12850386 Am J Hypertens, 2003
- RESULTS We demonstrated that interleukin 1beta IL 1beta, interleukin 1 receptor  [ **IL1R1** ]  2 IL 1R2 and interleukin 1 receptor  [ **IL1R1** ]  antagonist IL 1RA  [ **IL1RN** ]  genes are expressed in equine granulosa cells.  Ref: 12803652 Reprod Biol Endocrinol< MedlineTA>Reprod Biol Endocrinol, 2003
- To investigate whether the induction of proinflammatory cytokines contributed to the observe pathology in this disease, we infected rats with T. brucei and treated them with intracerebral infusion of the cytokine antagonists interleukin 1 receptor  [ **IL1R1** ]  antagonist IL 1ra  [ **IL1RN** ]  and or soluble type I receptor  [ **IL1R1** ]  of the tumor necrosis factor sTNFr1.  Ref: 12742658 J Neuroimmunol, 2003
